# American Academy of Dental Science

**Published:** 1896-11

**Authors:** 

**Affiliations:** American Academy Dental Science


### AMERICAN ACADEMY OF DENTAL SCIENCE.

The regular monthly meeting of the American Academy of Dental Science was held at Young's Hotel, Boston, Wednesday evening, April 1, 1856, at six o'clock, President Andrews in the chair.

A paper was read by Henry W. Gillett, D.M.D., of Newport, R. I.; subject, "Cataphoresis."

(For Dr. Gillett's paper, see page 704.)

Also, one by Philip W. Davis, S.B.; subject, "Electricity and its Application in Dentistry."

(For Mr. Davis's paper, see page 694.)

### DISCUSSION OF DR. GILLETT'S PAPER.

*Dr. Allen.*—I have been requested to relate my experience in the use of the "volt-selector," made by the Electro-Therapeutic

Company, of New York. Briefly stated, it seems capable of accomplishing all that is claimed for it as a means of obtunding sensitive dentine. Six weeks ago I purchased one of these instruments, and I am enabled to report favorably as to its obtunding power in a large percentage of the cases in which I have employed it. This fact alone would appear to commend its regular use in every case where the dentine is particularly sensitive, but, unfortunately, I find two serious obstacles to be overcome before it can possibly cover so large a field of usefulness: *First*, the time required to obtund the tooth ranges from twelve to thirty minutes, and often causes patient much fatigue in the process. *Secondly*, the current sometimes causes pain, even though very gradually applied, and occasionally the pain is so severe as to render its use impracticable.

Complete anæsthesia of the tooth may undoubtedly be obtained by cataphoresis, as I have demonstrated to my own satisfaction, provided sufficient time be given in the application; but as a mode of handling the many cases where an obtundent is indicated, I feel that the system has need of greater development and improvement in order to meet the needs of both the patient and the busy dentist.

Great credit is due Dr. Gillett for what he has done in bringing the subject of cataphoric anæsthesia in its present form before our profession, and should it finally develop into a system of general practice for the relief of our patients the world will owe him a debt of gratitude.

*President Andrews.*—I would like to call on Professor Brackett to give us an account of his experience with cataphoresis.

*Dr. Brackett.*—I consider it a privilege to give my testimony in regard to the efficiency of the apparatus which Dr. Gillett puts before us this evening. One of the earliest persons for whom I used it was a lady who has been my patient for more than twenty years, and who comes to me a distance of some seventy miles each year for attention to her teeth. She is the most sensitive one of two patients whom I have regularly for years permitted to take chloroform for the excavation of cavities. You all know how unbearable such work is for the occasional patient,—the few out of a thousand who seem to be totally unable to stand it. This lady's teeth are so sensitive that the attempt even to wipe out a cavity with a bit of bibulous paper literally makes her writhe in agony. The preparation of some small, simple cavities without any aid would cause her face to flush and the tears to roll down her cheeks, and the anguish was most extreme. She came to me in February for the annual fixing. She needed quite a number of fillings in a class of cavities, many of them cervical, from which she had suffered for a score of years, and by using this method I was enabled to work for her painlessly. After the first cavities had been prepared without the pain she had been accustomed to undergo, she said, "You take my blessings to Dr. Gillett for what he has done for me."

The system, the apparatus, the scheme, does accomplish that which is claimed for it, not without effort, not without time, and not with uniform facility; that is not to be expected; but that it does obtund sensitive dentine my experience has given ample evidence.

One of the questions which I would like to ask Dr. Gillett is about the relative impress upon the dentine of different degrees of density. It has been my inference, and it seems logical to suppose it to be so, that the extremely dense dentine is less readily susceptible to the influence of the benumbing agent than is dentine of a more vascular character. I think he will give us more positive information about that a little later.

In regard to the time required to obtain this effect, I have found it is necessary to spend a longer time than that spoken of by the essayist. With me, the length of the time of application has averaged about twenty minutes. In some cases it has taken twenty-five minutes to produce the full effect, and in other cases a much shorter time. I also differ from him in using a stronger solution of cocaine. In preparing the solution, it is my habit to place a little mass of the crystals on a tile and add a little water, not very much greater than the amount of cocaine, so that I have very likely a thirty- or forty-per-cent. solution. Of course, a person making use of this apparatus should have common sense expectations, and keep in mind certain simple conditions, which must be complied with in order that the greatest success may be obtained. It is quite natural that a person of nervous, sensitive, questioning organization should be somewhat apprehensive the first time it is used, but the difficulties we meet in this way are very simple, and just such as any reasonable mind would expect. If the results under such circumstances are not just what was hoped for, due allowance must be made. The time consumed in the preparation and filling of the most excruciating cavities, including the obtunding, the process may be shorter than would have been the case had the apparatus not been used, and the patient suffers very little if any discomfort. Sometimes there are a few hurts, as in making cuts in very sensitive dentine. Concerning these, one may choose between a second application of the current and the infliction of some suffering for a brief interval.

This does not appear to me an apparatus or a procedure to be used in every case, but for the cases of extreme sensitiveness, of which the one I first mentioned is a type, and for others not quite so severe, but in which there is unquestionably a great deal of pain in the preparation of cavities, it seems to me it is an invaluable blessing.

I feel that the work which Dr. Gillett has devoted to this, and the success which he has attained in bringing it to its present state of perfection, the pains he has taken to demonstrate practically that it will do these things, and his generosity in freely disseminating the information, should tend to bring honor to his name so long as dentistry is practised or its history is preserved.

*Dr. Williams.*—I have been very much gratified at listening to this paper, and I think we are indebted to Dr. Gillett for the clear manner in which he has placed this matter before us, and for the care which he has evidently taken in his investigations. The thought occurred to me that, in addition to making the application for the obtunding of pain, this method could be used for the introduction of antiseptics for saturating the roots of long devitalized teeth in order to get correct aseptic conditions. It strikes me as just the thing to make the proper application penetrate the substance of the dead root. You might use guaiacol or perhaps its carbonate, and by means of the electrical process thoroughly saturate the tubuli of the root with the antiseptic, and be reasonably sure of its being done.

*Dr. Wilson.*—I would like to ask Dr. Gillett if he has ever used this apparatus in Boston, and whether he found any difference between the current here and in Newport?

*Dr. Gillett.*—I used it at Dr. Cooke's office the other day in a clinic, but it would be impossible to make any comparative statement from that trial of it because I was paying attention to a good many different things at once. It is a fact that a current which is very irregular will be exceedingly unpleasant to any patient who is markedly sensitive to electricity; the patients will feel surges of pain as the current increases or diminishes. This irregularity may occur from different causes. You will notice it occasionally in wet and stormy weather, where the current is conducted by overhead wires.

*Dr. Smith.*—I would like to ask Dr. Gillett in what per cent. of cases the application has been absolutely without pain?

*Dr. Gillett.*—I do not know that I can answer that question accurately, because I am in the habit of pushing the application only to such a degree as the patient willingly stands. When I have tried to make the operation entirely painless, I have been successful, and I think it can be done in every case if you take time enough to do it.

If you have an assistant to make the application for you, or even give it into the hands of the patients themselves, which is a perfectly feasible thing to do, all the time desired may be taken for an application, and you can then go ahead and do the operation painlessly. If you can devise a way of taking the necessary time for the application it could be made absolutely painless in ninety-nine or perhaps one hundred per cent. of cases.

*Dr. Wilson.*—I wish to know if Dr. Gillett is willing to attempt to anæsthetize the teeth that we have occasionally where the pulp is not exposed, but the tooth is aching violently?

*Dr. Gillett.*—You mean would I have the confidence to attempt to anæsthetize the pulp in such a case? I have done so repeatedly. I doubt if it is possible to fully anæsthetize the pulp with cocaine through a thick layer of dentine, and I also doubt if it is possible to anæsthetize *every* exposed pulp. I have anæsthetized exposed pulps when they were not very vascular and not very large,—for instance, in laterals, where the exposure was comparatively large. In those cases you can feed the cocaine in rapidly enough to anæsthetize the pulp without causing much pain, and in everything except a violently inflamed pulp you may be able to remove the pulp entirely. It is my habit, however, to just benumb the surface, and then use the syringe for injecting the cocaine, as recommended by Dr. Briggs. I can do that in five minutes, while the other method takes perhaps half an hour.

*Dr. Meriam.*—Is it possible to anæsthetize through a crown cavity and afterwards excavate on a buccal in the same tooth?

*Dr. Gillett.*—I have never been able to anæsthetize one cavity through another. I think if you continued the application long enough you might eventually produce that effect by partially anæsthetizing the pulp itself. It is doubtful, however, if such long applications are desirable.

*Dr. Clapp.*—We should be most grateful to Dr. Gillett for putting this matter before us in a manner to be comprehended by our uninstructed minds. It has been made remarkably clear in spite of the fact that we are dealing with a science of which few of us have much knowledge. I wanted to ask him a couple of questions:

Suppose we have a cavity in which is an old filling, must it be removed or insulated before beginning the anæsthesia? I should also like to know whether this effect extends to the gum or surrounding tissues?

*Dr. Gillett.*—It would not extend appreciably through the tooth to the gum with the rubber in place.

Cavities that extend to the edge of fillings sometimes present difficulties. These may be surmounted in two ways. You may find that your patient is not so sensitive to the effects of the current but that he can bear the amount necessary to anæsthetize the territory desired, even if it does make connection with the filling. The objection to letting the current go through the filling is not that it does any harm to the filling, but that the patient often finds it unbearable. The other method is to cut away what you can of the filling, and then put on a little layer of varnish, gutta-percha, or other insulating material, and apply your current at the point you wish to anæsthetize. In the case of a filling that extends down to the gum, and it is exceedingly difficult to get *any* instrument down there without causing pain, it is rather hopeless to expect to use this method.

*Dr. Clapp.*—Will a coating of sandarac varnish be sufficient to insulate a filling?

*Dr. Gillett.*—I should expect it to be sufficient.

*Dr. Clapp.*—In regard to our irregular current here in Boston. I have noticed that my lights in the evening are exceedingly irregular. At certain times they go up and down with a very regular variation. I am inclined to think that in the daytime the current would not be so irregular, because there is less demand on the electric light company.

*Dr. Gillett.*—The difficulties are not from slight irregularities, but from interruptions of the current. It would be undesirable to use a source of supply where the current is interrupted or diverted almost entirely for a short time. A gradual increasing or decreasing of the current would not make so much difference.

*Dr. Payne.*—I would ask Dr. Gillett if there are any after-effects from this method,—that is, after the cocaine has been used by cataphoresis and the cavity filled with gold, if the tooth is hypersensitive to heat and cold?

*Dr. Gillett.*—So far as I have been able to judge, the after-effects do not differ from what they would be in the case of the same cavity filled without the use of electricity.

I have watched carefully myself, and have asked some of my most intelligent patients who have had it used and know just what has been done, if they would watch the teeth where this method had been used, and I have never been able to get any evidence of effects that I could distinguish from the after-effects of fillings put in in the ordinary manner. I have found absolutely no constitutional or systemic effects.

*Dr. Taft.*—I would like to know what Dr. Gillett's experience has been in the treatment of sensitive cavities in children. They are among the most trying cases we have to deal with.

*Dr. Gillett.*—Of course, you could not use it with the younger children, because they will not keep still long enough, and the limitation is about the age when you can use the rubber dam. It can be used in approximal cavities in bicuspids and first molars, at the time when the teeth are so very sensitive,—say, between ten and thirteen. I used it in putting in a filling for a boy of twelve, who went to sleep under it the first time it was used. I knew his teeth to be very sensitive, because on previous occasions it had taken just about all his courage to allow me to prepare a cavity.

*Dr. Werner.*—I would like to ask Dr. Gillett in what category of success he would place the operation which was performed in Dr. Cooke's office last Thursday?

*Dr. Gillett.*—Fairly successful for a clinic. The result was not as satisfactory to the patient as if he had told me at the time that he felt the pain. I understand that he afterwards made the statement that it did hurt him considerably. If he had said so at the time I would have made a longer and more gradual application.

*Dr. Werner.*—He told me that he suffered a fair and square toothache during the whole time of the application. Do you think if you had applied it fifteen minutes longer the pain would have been less or none?

*Dr. Gillett.*—He might have felt a sensation, but nothing like what it would have been if the application were not made. It is an application that I have made repeatedly with the best of success in the great majority of cases where I have used it. I have tried it in the cases of nervous children who did not know that anything out of the ordinary was being done, and have operated for them without causing them pain. I have tried it with sensitive women, and there are no patients who will let you know quicker when you are hurting them than they.

*Dr. Eames.*—I would like to ask if it is not true, especially in the form of apparatus which is connected with the chair, if you do not have to guard against the patient unsuspectingly taking the hand off and breaking the connection? Also, your electrode, which is applied to the cavity,—is it not necessary to guard against its removal while the current is on?

*Dr. Gillett.*—It will make you jump if you break the connection by moving an electrode. You will get more irritation by moving the positive electrode—the one on the tooth—than you will by moving the negative electrode. I have had very little trouble in this line,—in fact, I habitually give the negative electrode to my assistant, and I think no more about it. When I first began I found no difficulty in putting it into the hands of the patients themselves, only in such cases I took a little more pains to see that they did not get scared at something and drop the electrode.

*Dr. Eames.*—The reason I spoke about it is that I have used the dry chloride of silver battery for other purposes,—for instance, electrolysis,—and I have noticed that when I trust the electrode to the patient sometimes they take it off, and it gives them a little shock.

There is one more question I wish to ask the essayist,—May we not infer that a strong solution of cocaine is necessary, as you mention high percentages?

*Dr. Gillett.*—I have not worked with anything less than fifteen per cent. My impression is that the stronger solutions work better up to a certain point, but I think thirty per cent. strong enough.

*Dr. Eames.*—I wish also to remark that it seems to me that the milliampère meter ought always to be used. It is simply criminal not to use it in view of the varying degrees of resistance and susceptibility in different persons.

*Dr. Gillett.*—There is a difference of fully one hundred per cent., —that is to say, you may get one milliampère through a tooth with a certain force of current, while in other teeth you could get two milliampères with the same force.

*Dr. Stevens.*—Some one asked the question about using a larger electrode in a large cavity, and I would offer this suggestion, that you might get a larger area by placing a platinum disk on the cotton and applying the electrode to the disk.

*President Andrews.*—If no other members wish to speak I will ask Dr. Gillett if he will close the subject.

*Dr. Gillett.*—I will try and be brief in what I have to say. To Dr. Eames I would say that I have never seen any after-effects whatever from the current, so far as I have followed the matter. Dr. Marshall presented two or three years ago a very interesting paper before the American Dental Association, referring to the use of the galvanic current to subdue irritated conditions and to stimulate sluggish conditions of the pulp. I have not followed out that line especially, and consequently have not noted such effects on the pulp. I assume that Dr. Marshall's statements are correct.

As the other gentlemen who have used the method stated, the main objection is the time consumed. I do not attempt to use it in cases where there is only a little sensitiveness to be overcome,—it isn't worth while. There are a great many cavities where some other method will be more prompt; there are a great many cavities where the patients would not be willing to have you spend the necessary amount of time for the sake of preventing the little pain they would have to endure. I have repeatedly said that it is not a method to be used in every operation about the teeth, even when you know you will have to cause pain. The typical case is where the cavities are so sensitive that to operate in them causes the most extreme pain, and all but prostrates the patient. Those cases, so far as my experience of a year has gone, can be placed under control with comparative ease. It is my hope, as I outlined in the paper, that we may be able in the future, with some other drugs, to cut down the time necessary for anæsthetizing. If guaiacol proves to be a feasible drug, the time may be reduced to one-half or two-thirds of the time required for cocaine. As I suggested, in replying to Dr. Smith's question, one of the means of solving the problem is to have an assistant who is capable of making the application, and thus save your own time, or, as I also suggested, it seems perfectly feasible for the patient to make the application for himself,—I believe that can be done.

There are some cases where it is very difficult to make the application on account of sensitiveness of the patients to the electric current.

Dr. Brackett stated that the time required in his cases was about twenty minutes to half an hour. Without counting up positively, I should say that a dozen or fifteen cases are as many as have accumulated in a year's use where I have been obliged to continue the application longer than twenty minutes, but I had a very few cases where I thought it wise to extend it to a full half-hour. One case was that of a very large crown approximal cavity, and having a previous acquaintance with the sensitiveness of the patient's teeth, I know that without the assistance of this method I could not have prepared that cavity, the patient simply could not have borne it. After a half-hour's application, I cut that cavity all I wanted, and put in a gold filling that will stay, instead of making a cement filling needing renewal every year.

In that case it made an operation possible that was simply impossible before. In reply to Dr. Brackett's question as to dense dentine being less easily obtunded, such is a fact, and the reason is perfectly plain,—there is less animal tissue there to take up the cocaine or to conduct the current.

With regard to what Dr. Williams suggests, the penetration of roots for their disinfection,—that is one of the possible and also one of the probable uses of the method. There is also a possibility of its being of service to us in the treatment of obstinate abscesses, and its possible value for use on ulcerated surfaces in pyorrhœa alveolaris has been just touched upon in one of the published articles by Morton. There is, it seems to me, a probability of overcoming that very difficult condition of affairs where you have exposed dentine on the surfaces of molars. Now, it should be entirely feasible to anæsthetize that tissue, and then apply the actual cautery, or perhaps to drive the nitrate of silver or some other cauterizing agent directly into that hypersensitive dentine. There are numerous other uses to which it can be put, which will suggest themselves to the operator, in his daily work.

I want to reiterate that the time is the main objection, but I think that objection will eventually be overcome. At present it is not a method for every case; it is a method by which I have been able to overcome the difficulties of extremely sensitive cases, and do it in a manner which has apparently been very satisfactory to my patients and certainly so to myself.

*Dr. Brackett.*—Referring again to the matter of time, I will say that my most frequent use of it ranged between fifteen and twenty-five minutes, though, as I stated, there were cases where I was obliged to continue the application for half an hour.

*Dr Bradley.*—I think I voice the sentiments of the Academy in saying we are very much indebted to Dr. Gillett for his very excellent paper, not only for his explanation of the special uses of the milliampère meter, but for the fact that he has told us about it in such a way as to make it plain to those of us who are not acquainted with the terms used in electricity; therefore, I desire to offer a motion that we extend to him a vote of thanks for his paper this evening.

Unanimously voted.

William H. Potter, D.M.D.,
*Editor American Academy Dental Science.*

